# Osseous choristoma of the tongue: two case reports

**DOI:** 10.1186/s13256-016-0840-8

**Published:** 2016-03-17

**Authors:** Bhoj Raj Adhikari, Jun Sato, Tetsuro Morikawa, June Obara-Itoh, Masafumi Utsunomiya, Fumiya Harada, Takatoshi Chujo, Rie Takai, Koki Yoshida, Michiko Nishimura, Mamata Shakya, Hiroki Nagayasu, Yoshihiro Abiko

**Affiliations:** 1 Division of Oral Medicine and Pathology, Department of Human Biology and Pathophysiology, School of Dentistry, Health Sciences of University of Hokkaido, 1757 Kanazawa Ishikari-Tobetsu, Hokkaido, 061-0293 Japan; 2 Division of Oral Maxillofacial Surgery, Department of Human Biology and Pathophysiology, School of Dentistry, Health Sciences of University of Hokkaido, 1757 Kanazawa Ishikari-Tobetsu, Hokkaido, 061-0293 Japan

**Keywords:** Maxillofacial region, Oral mucosa, Osseous choristoma, Osteoma, Tongue

## Abstract

**Background:**

Osseous choristoma is a very rare, benign lesion in the maxillofacial region. It appears as a benign mass of normally matured bony tissue covered by the normal epithelium of the tongue. It is usually seen in front of the foramen cecum of the tongue. Surgical excision is the treatment of choice with an excellent prognosis and there have been very few cases of recurrence.

**Case presentation:**

Here we present two cases of osseous choristoma on the dorsum of the tongue. Case 1 was a 15-year-old Japanese girl who presented with a painless but gradually growing swelling on the dorsum of her tongue approximately 1 year before her admission. Case 2 was a 21-year-old Japanese woman with a complaint of pain in the lower left, posterior side of her mouth. Histological findings showed that both lesions were composed of well-organized, mature, compact bone beneath the oral mucosal membrane. Subsequent to simple surgical excision, no recurrence of the lesions was observed after the follow-up period. Previous literatures have proposed both malformation and trauma hypotheses as the etiopathologies of osseous choristoma. However, the histopathological findings of the two cases in the present study do not support the trauma hypothesis.

**Conclusions:**

Although osseous choristoma is clinically a benign condition, the underlying histopathological processes are important. The outcome of aberrant formation of calcified tissue in the vicinity of vital structures such as nerves and blood vessels may be of clinical significance.

## Background

Osseous lesion of the tongue is a rare entity and was originally reported as a lingual osteoma [1]. Since the biological behavior of the lesion did not meet the criteria of a tumor, the term osseous choristoma was proposed and is currently widely used for this lesion [2]. Choristoma is defined as a growth of normal tissues in an ectopic location [3, 4]. So far, only 67 cases of osseous choristoma of the tongue have been reported in the literature [5]. Here we present two additional cases of lesion in the tongue and discuss the epidemiology, clinical presentation, and debatable pathogenesis of this disease.

## Case presentation

### Case 1

**Fig. 1:**
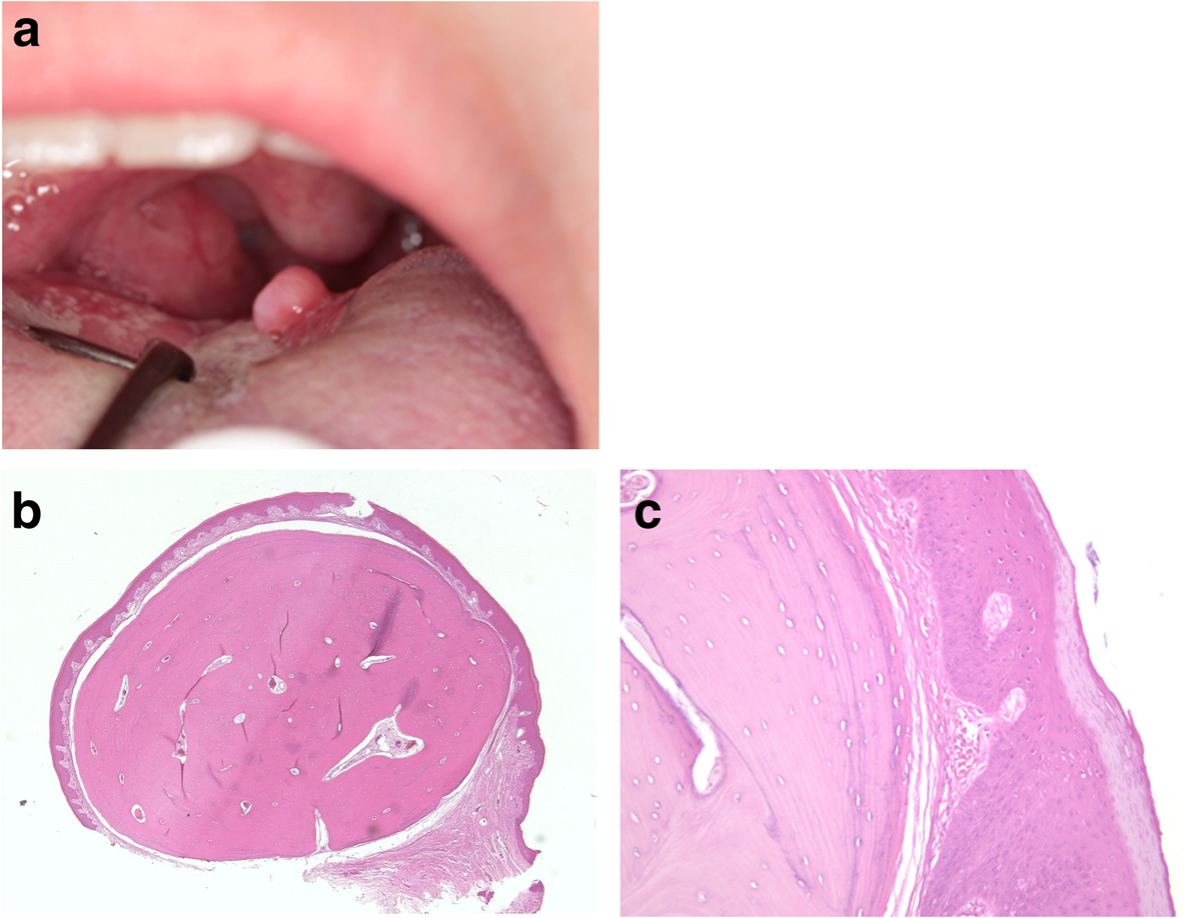
Clinical and histological appearance of Case 1. **a** A tumor mass on the left side of the posterior dorsum of the patient’s tongue, covered by healthy looking oral mucosa. **b** On histological examination, a nodule of matured bone surrounded by fibrous stroma and lined with stratified squamous epithelium was noted. **c** Higher magnification of the tumor showing matured bone with lamella structures surrounded by fibrous connective tissue. Although no identical osteoblastic or osteoclastic reactions are observed, osteocytes can be seen within the lacunae

A 15-year-old Japanese girl was referred to our out-patient department (OPD) with a chief complaint of discomfort in her throat. She had noticed a painless but gradually growing swelling on the dorsum of her tongue approximately 1 year before her admission. Her past medical history revealed that she had taken iron-containing medications for a short period of time; however, there was no documentation of her medical condition. An intra-oral clinical examination revealed the presence of a pedunculated well-circumscribed swelling, measuring 5 mm, on the left side of the posterior dorsum of her tongue, near the foramen cecum. It was covered by healthy looking oral mucosa (Fig. 1a), and felt elastic hard and granular on palpation, suggesting a diagnosis of fibroma. An excisional biopsy was performed under local anesthesia, and the tissue was immediately placed in 10 % formalin for fixation. Clinical evidence of epithelialization with uneventful healing of the surgical wound was observed when the patient was reviewed on the 11th day. She had remained free of the disease 5 months after the surgery. Histological findings showed that the lesion was composed of well-organized, mature, compact bone with lamellar structures beneath the mucosal membrane, and lined by orthokeratinized stratified squamous epithelium (Fig. 1b, c).

### Case 2

A 21-year-old Japanese woman visited our OPD with a chief complaint of pain in the lower left, posterior side of her mouth. An intra-oral clinical examination revealed the presence of partially impacted, lower third molars bilaterally, both of which required surgical extraction. She did not have any other abnormal, medical or surgical history. Further clinical examination revealed the presence of a pedunculated well-circumscribed swelling, measuring 5 mm on the right side of the posterior dorsum of her tongue, near the foramen cecum. The lesion was lined by a thin normal-looking mucosa, and was bony hard on palpation. The lesion was symptomless and accidentally discovered during her oral cavity examination. Her chief complaint was addressed first, and the tongue lesion was planned for surgical excision at a later period. An excisional biopsy of the lesion was performed under local anesthesia, and the tissue was immediately placed in 10 % formalin for fixation. She was reviewed on the second and seventh day during which her healing was found to be uneventful. She was kept under observation and recalled 1 month, 6 months, and 2 years after surgery, and was found to remain free of the disease at the end of the follow-up period.

**Fig. 2:**
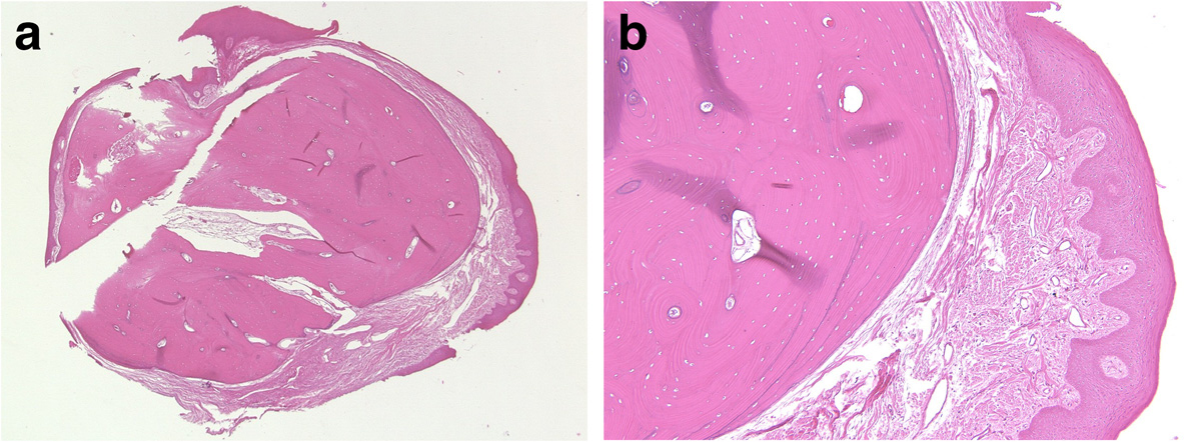
Histological appearance of Case 2. **a** A nodule of matured bone surrounded by fibrous stroma and lined with stratified squamous epithelium. **b** Higher magnification of the tumor showing matured bone consisting of osteon-like structures and surrounded by fibrous connective tissue. Osteocytes are seen in the lacunae

Histopathology showed that the lesion was composed of well-organized, mature, compact bone with lamellar structures beneath the mucosal membrane, and was lined by parakeratinized, stratified, squamous epithelium (Fig. 2a, b).

## Discussion

Osseous choristoma of the tongue is extremely rare with only 67 cases reported in the English literature so far [5]. Here we have described two additional cases of osseous choristoma; the clinical and histological findings obtained from the two cases enabled us to reach the final diagnosis. The age of patients with osseous choristoma has been reported to range from 5 to 73 years with a mean age of 28.7 years [5]. The lesion is more prevalent in females than in males [4, 5]. The tumor is located in the posterior third of the tongue near the foramen cecum, most commonly around the circumvallate papilla, and sometimes at the lateral border or middle thirds of the tongue [5]. The size of the lesion varies from 3 mm to 5 cm [5], and the duration ranges from 3 days to 50 years [4]. The lesions present as a hard mass and may be either pedunculated or sessile. The mucosa covering the lesion usually appears as normal epithelial mucosa, although ulcerated mucosal covering has also been reported [4]. The clinical findings including age, gender, size and macroscopic appearance of the two cases in the present study are similar to those reported earlier.

The etiopathology of osseous choristoma is still unknown. Two different hypotheses have been proposed; whereas one suggests that this is a type of malformation, the other concludes that it is formed as a result of trauma or chronic irritation [6]. According to the malformation hypothesis, the lesion arises at the line of fusion of the first and third brachial arches between the anterior two-thirds and posterior one-third of the tongue [1]. This hypothesis is consistent with the knowledge that certain normal osseous structures such as the incus and malleus, and the hyoid bone are derived from the first and third brachial arches, respectively. Pluripotent cells originating from these arches might have been trapped within this region and subsequently undergone ossification resulting in the development of osseous choristoma [6]. In another study, the foramen caecum has been stated as the site of development of the anlage of the thyroid gland during embryonic life [1]. Remnants of the undescended intraglossal thyroid tissue can lead to unusual osseous proliferation later in life [7]. The trauma hypothesis, on the other hand, indicates that the posterior third of the tongue is highly susceptible to trauma and irritation, and an osseous lesion on the tongue may represent a reactive or posttraumatic center of ossification. The occurrence of “myositis ossificans” in other muscles may support this hypothesis [8]. However, some studies have demonstrated normal bone formation in these lesions, in contrast to the irregularly formed bone lacking the Haversian system seen during traumatic ossification [9]. Histological findings revealed normally formed bone in both cases in the present study and might therefore not support the trauma hypothesis.

The differential diagnosis of a protruding mass in the tongue can also be based on its location. When the lesion is on the dorsum of the tongue near the foramen caecum, it can be considered a benign tumor such as hemangioma, lymphangioma, teratoma, hamartoma, leiomyoma, thyroglossal duct cyst, lingual thyroid, mucocele, and pyogenic granuloma, or a malignant tumor such as rhabdomyosarcoma and epidermoid carcinoma. Lesions found on the margins of the tongue may be diagnosed as a traumatic neuroma, neurofibroma, schwannoma, fibroma, or cartilaginous choristoma. Pyogenic granulomas, mucoceles and cartilaginous choristomas have a greater predilection for the anterior region of the tongue. Other differential diagnoses include vascular malformation, fibroma, papilloma, salivary gland mixed tumor, osteochondroma, and other neoplasms such as osteogenic sarcoma or squamous cell carcinoma [5]. Hamartoma is a similar morphological entity which is often confused with choristoma [3]. Choristoma is a histologically normal tissue proliferation of a type that is normally not found in the anatomic site. On the other hand, hamartoma is a disorganized proliferation of mature tissues, composed of elements that are normally found in the specific location in which it develops, often with one predominating element [3, 10]. The diagnosis of a lesion would therefore depend on its anatomic site. Since there is no bone tissue in the tongue, our cases were diagnosed as choristoma. Case 1 was initially diagnosed as a fibroma; the definite diagnosis was reached after histological examination of the lesion.

Surgical resection is the most common treatment for osseous choristomas [4, 5], although potassium titanyl phosphate (KTP) laser has been used to excise the lesions [11]. The post-surgical prognosis of lingual osseous choristomas is excellent, and only two cases of recurrence have been reported so far [12]. In the present study, neither of the two cases showed any sign of recurrence.

## Conclusions

This study presents two rare cases of osseous choristoma in the posterior part of the dorsum of the tongue, and adds to the existing knowledge of the condition.

## Consent

Written informed consent was obtained from patient 1’s legal guardian and written informed consent was obtained from patient 2 for the publication of this case report and the accompanying images. Copies of the written consents are available for review by the Editor-in-Chief of this journal.
